# Pneumonia: Its Supposed Connection, Pathological and Etiological, with Autumnal Fevers; Including an Inquiry into the Existence and Morbid Agency of Malaria

**Published:** 1854-04

**Authors:** 


ART. IX.—*Pneumonia: its supposed connection, pathological and etiological, with Autumnal Fevers; including an inquiry into the existence and morbid agency of Malaria.* By R. La Roche, M. D., Member of the American Philosophical Society, etc., etc., etc. Philadelphia: Blanchard & Lea. 1854.

Very many of those who peruse the above must be familiar with the fact that Dr. La Roche has written previously on malaria. They will recollect the care and labor with which those monographs were prepared, and the vast number of authorities whom the talented and laborious author had consulted.

We confess to a little surprise on noticing the word "Pneumonia" at the head of the title-page. Malaria has been his specialty; to it he has devoted the labors and observation of a prolonged professional life, and we should have supposed that when Dr. La Roche published a volume of so respectable a size as this, it would have been upon his favorite subject. But as he himself says "*L' homme propose, et Dieu dispose;*" and the Dr. comes out at last with a goodly volume upon the connection of pneumonia and malaria.

It seems to us that this effort partakes a little of the character of a kick at a dead lion. Here is learning and research enough to have solved the riddle of the Sphynx, devoted to this "supposed" connection. Not that the author supposes any such improper intimacy. On the contrary, he is convinced that pneumonia is a disease *sui generis*, without even the propinquity of an Irish cousinship to autumnal fevers. Like all Frenchmen (we suppose Dr. La Roche to be of Huguenot extraction) he gives us, first, "l' historique." He goes back and wakes up Laucisi, Sydenham, and Cleghorn, and from that point of departure he brings us comfortably adown the centuries, telling us what every body said upon the subject up to this year of our Lord 1854.

It seems that the doctrine which assigns a common causation to intermittents and pneumonias is still extant, but we had no idea that it existed in such an important form, as to demand so ponderous a weapon for its overthrow as this volume furnishes. We recollect to have heard, in our student days, of bilious pneumonias and pleurisies, (a nomenclature derived among the jaundiced pioneers of the early west,) but we had lived so long without hearing from it, that we supposed it dead, or living only among the ranks of the "monumentals," those good conservative souls who run not astray after every (*or any*) new thing.

So when we found five hundred and two pages devoted by a really able author to the discomfiture of this moss-grown and antiquated theory, we had that same feeling of sympathy for the erring and the lost, that we do every year, when all our political parties fire a broadside of resolutions at the United States Bank.

And it seemed to us not only unfair, but useless, to attempt to disturb the quiet rest of these old monumentals. They will not read La Roche on pneumonia and malaria—if they do they will not believe it—it is hardly probable that they will even see this notice, for they do not take the Journal.

Let not our readers suppose that we wish to decry this book; it has its merits, and some of them are of high order. Its style is admirable: the very model of good medical style; classic, but not so severely so as to be altogether unwarmed by the glow of happy expression, and pithy illustration. Its facts *are* facts; it exhausts the subject, and leaves no stone of argument unturned.

We have yet to mention that which gives the highest value to the book. It has been fashionable of late (under the leadership of Dr. Dundas and others) to decry the very existence of malarial poison. Men in high places have (reasoning from final causes only) asserted that the time-honored belief in marsh-miasmata was an error.

No man in the country is better qualified to refute this error (it is an error, for our eyes have seen, and our nose has smelt, malaria) than Dr. La Roche. And he does refute it. If a long array of facts, well stated and convincing, and backed up by clear and logical argument, can reinstate the profession in its faith in the existence of malaria, this book must do it.

We only wish that malaria had been made the prominent, and pneumonia the secondary subject in this essay. So few believe in any connection of this cause and effect, that we naturally ask of such a labored argument, "*cui bono?*" But the matter of malaria is just now more important than ever; it is as largely discussed, and the resulting belief of the profession must have great influence upon the establishment of sanitary measures in city and country. H.

For sale by Miller, Orton & Mulligan.

